# *Leishmania* Sexual Reproductive Strategies as Resolved through Computational Methods Designed for Aneuploid Genomes

**DOI:** 10.3390/genes12020167

**Published:** 2021-01-26

**Authors:** Jahangheer S. Shaik, Deborah E. Dobson, David L. Sacks, Stephen M. Beverley

**Affiliations:** 1 Discovery Bioinformatics, NextCure Inc., Beltsville, MD 20705, USA; 2 Department of Molecular Microbiology, School of Medicine, Washington University, St. Louis, MO 63110, USA; dedobson@wustl.edu (D.E.D.); stephen.beverley@wustl.edu (S.M.B.); 3 Laboratory of Parasitic Diseases, National Institute of Allergy and Infectious Diseases, National Institutes of Health, Bethesda, MD 20814, USA; dsacks@niaid.nih.gov

**Keywords:** aneuploidy, meiosis, next-generation sequencing, bioinformatics, chromosomal inheritance, *Leishmania*, clonality and sexuality

## Abstract

A cryptic sexual reproductive cycle in *Leishmania* has been inferred through population genetic studies revealing the presence of hybrid genotypes in natural isolates, with attempts made to decipher sexual strategies by studying complex chromosomal inheritance patterns. A more informative approach is to study the products of controlled, laboratory-based experiments where known strains or species are crossed in the sand fly vector to generate hybrid progeny. These hybrids can be subsequently studied through high resolution sequencing technologies and software suites such as PAINT that disclose inheritance patterns including ploidies, parental chromosome contributions and recombinations, all of which can inform the sexual strategy. In this work, we discuss the computational methods in PAINT that can be used to interpret the sexual strategies adopted specifically by aneuploid organisms and summarize how PAINT has been applied to the analysis of experimental hybrids to reveal meiosis-like sexual recombination in *Leishmania*.

## 1. Introduction

### 1.1. Clonality vs. Sexuality

Protozoan parasites of genus *Leishmania* are responsible for Leishmaniasis, which are vector borne diseases infecting humans and domestic animals. In humans, *Leishmania* infections can manifest as cutaneous, mucocutaneous, and visceral disease forms that reflect the extensive strain and species diversity of the genus. *Leishmania* parasites have a complex life cycle, alternating between asexually reproducing extracellular promastigotes and intracellular amastigotes within the sand fly midgut and host macrophages, respectively. A nonobligatory and cryptic sexual reproductive cycle is now known to accompany parasite development in the sand fly vector [1,2], and the current clonality vs. sexuality debate pertains mainly to the frequency and mode of genetic exchange, and its impact on population structure.

The predominant clonal evolution (PCE) theory, proposed in the 1990s for *Leishmania* and related organisms [3,4], argues that *Leishmania* are mainly clonal with only occasional bouts of recombination that fail to break the predominant clonal population structure. PCE also proposes that selfing, a sexual process involving closely related organisms [5], is a special case of clonality where the progeny are genetically identical to the parents [3]. The population genetics studies supporting clonality, however, often remain unclear because of the limited number of markers studied or due to improper sampling [6]. The issue of limited markers has been addressed to some extent using multilocus sequence typing and microsatellite markers [7,8,9], and especially due to the advent of Next-generation sequencing (NGS) technologies [10,11]. Improper sampling, where the strains being studied are neither from specific geographical regions nor collected at comparable times, remain an issue because it violates the assumptions made by the computational algorithms that predict the population structure [12]. Furthermore, *Leishmania* genomes exhibit constitutive aneuploidy, including mosaic aneuploidy, where chromosome numbers may vary from cell to cell [13,14], while the computational tools used to study them are built for diploid genomes. Alternate computational strategies coupled with improved next-generation profiling technologies promise to provide more insights into the mode of sex and its frequency in *Leishmania*.

### 1.2. Mosaic Aneuploidy and Parasexuality

**Figure 1:**
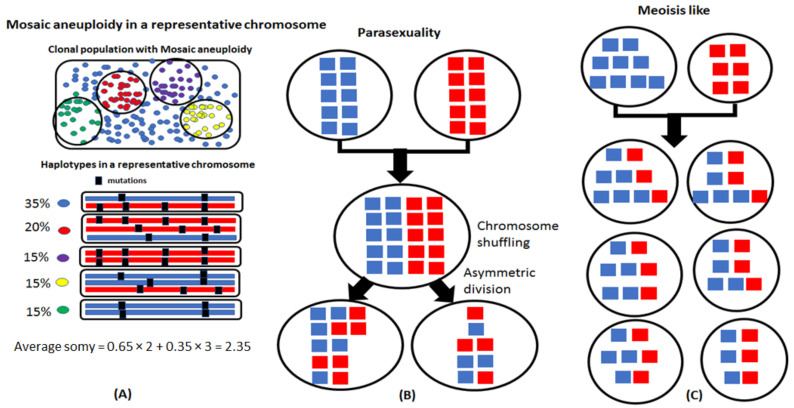
Possible sexual strategies in *Leishmania*. (**A**) Mosaic aneuploidy is a common feature of *Leishmania* where different cells in a population can have different chromosome copy numbers. Haplotypes of a hypothetical chromosome and their occurrence in a population are shown. Heterozygosity could be a consequence of mosaic aneuploidy and not strictly due to the allelic differences between homologous chromosomes. (**B**) A parasexual process involves fusion of parental chromosomes followed by random shuffling and reductional division. The products of parasexuality resemble those of meiosis. However, a parasexual process results in frequent asymmetric allocation of chromosomes and few homologous recombinations in contrast to meiosis. (**C**) Meiosis results in predictable chromosomal allocations. When both the parents are disomic, hybrids are disomic due to inheritance of a chromosome each from both parents. A trisomic parent can share either one or two chromosomes, while a wide range of inheritance possibilities occur when one or both parents are tetrasomic.

*Leishmania* traditionally were considered mainly diploid and aneuploidy was associated with certain chromosomes depending on the species and strain [15]. However, based on studies employing fluorescent in situ hybridization (FISH), aneuploidy seems to be the norm, at least for promastigote stages growing in culture [14]. A few chromosomes that were studied in multiple species exhibited some mosaicism in ploidy and the cells routinely showed asymmetric chromosomal allotments to the dividing nuclei during interphase and mitosis (Figure 1A). This genomic plasticity provides *Leishmania* with the potential to generate phenotypic diversity, allowing it to adapt to different environments [16]. Aneuploidy can also have interesting ramifications on genome structure dynamics, such as loss of heterozygosities due to clonal asymmetric division and gain of heterozygosities due to hybridizations involving cells with homozygous and alternate genotypes during automixis.

Traditional meiosis is usually associated with stable diploid genomes where reductional division of parental cells into haploid gametes is accompanied by chromosomal recombination and random assortment, followed by cell fusion to form the hybrids. For the aneuploid genomes of *Leishmania*, instead of a precise reductional division into gametic cells, a parasexual process was argued involving fusion of parental cells followed by chromosomal shuffling and concerted chromosome loss [17] (Figure 1B). The observation of cell fusions in promastigotes using video microscopy is consistent with a parasexual process [18]. Recombinations between homologous chromosomes are not a routine phenomenon in parasexuality but have been observed in low frequency during the parasexual cycle in *Candida albicans* [19].

### 1.3. Natural and Experimental Hybrids in Leishmania

Natural *Leishmania* isolates have mostly shown little heterozygosity, which can be explained by limited outcrossing, but can also be attributed to selfing [5]. In addition, heterozygosities may fall under the limits of detection due to their unconventional frequencies in an aneuploid genome. Nonetheless, many reports offer clear evidence of hybridizations within and between different *Leishmania* species based on the presence of heterozygous alleles [20,21,22,23,24,25,26,27,28]. Hybrids have been detected using a few isolated markers, which may be adequate if the hybridization event is recent and occurs between two different genotypes. However, in cases where extensive inbreeding or rare mating has occurred, dense markers may be needed for proper admixture detection. For example, whole genome sequencing (WGS)-based analysis of 11 *L. infantum* strains from Turkey was able to detect a genome-wide patchy heterozygosity pattern involving two different genotypes [11]. This pattern suggested a single outcross hybridization event followed by clonal reproduction and inbreeding. The presence of genetic markers from two different strains/species is not by itself a proof of meiotic recombination and can also be explained by a parasexual process [17]. Controlled experiments that generate hybrids in the laboratory using known parental lines provide ways to elucidate these processes in *Leishmania*.

Experimental hybrids were first generated using two different strains of *L. major* by introducing two drug resistance markers into the parental lines, coinfecting sand flies, and selecting for double-drug resistant hybrids [1]. All the hybrid clones inherited a full set of allelic markers from both the parents and were either diploid or triploid. The sexual competency of additional *L. major* strains was subsequently demonstrated in both natural and unnatural vectors [29], and cross-species genetic exchange between a visceral and cutaneous strain of *Leishmania* was experimentally demonstrated [30]. These experiments suggested that neither the parasite nor the sand fly vector species are limitations to sex in *Leishmania*. Based on analysis of high-resolution whole genome sequencing data performed using concepts from PAINT such as determining somies, finding loci where parental lines were homozygous and different from each other, estimating the frequencies of those parental alleles in hybrids to determine parental contributions and visually painting those contributions (Therefore the name PAINT for the software) to learn not only about the contributions but also events such as loss of heterozygosities in 44 experimental hybrids, our recently published study argues in favor of meiosis-like sexual recombination in *Leishmania* [31]. In this work, we discuss the bioinformatics strategies incorporated into a software suite PAINT that were employed for the analysis and interpretation of our experimental hybrid data.

## 2. Methods

### 2.1. PAINT, a Comprehensive Tool to Decipher Sexual Strategies in Leishmania

**Figure 2:**
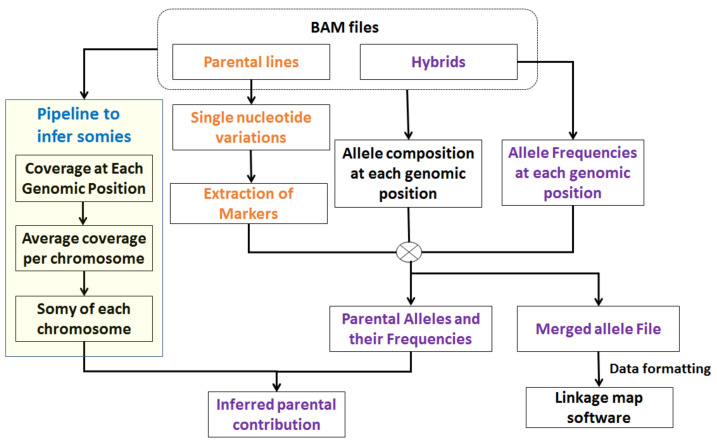
Overview of PAINT pipeline. The input to PAINT pipeline is a sorted binary alignment map (bam) or a sorted sequence alignment map (SAM) file. PAINT determines the chromosomal somies based on average chromosome coverage and ploidy of the organism. PAINT finds loci where parental lines are homozygous different and employs them as markers. It determines the parental allele frequencies and in conjunction with chromosomal somies infers parental contributions. It also compiles the allele information from all the samples at the selected markers and summarizes them into formats that can be used as inputs to other softwares.

The advent of NGS technologies has enabled probing into complex questions by leveraging a high-resolution snapshot of the genome obtained using high-throughput sequencers such as Illumina Hi-seq machine. The first routine step in most NGS-based analyses is to align the read sequences to a reference genome using alignment algorithms such as Novoalign [32]. The raw alignments to the genome in sequence alignment map (SAM) or binary alignment map (BAM) sorted by genomic position are input to PAINT (see Figure 2). PAINT then quantifies the somies based on the read alignments, extracts relevant markers, finds the nucleotide compositions and allele frequencies at the selected markers, and quantifies parental chromosomal inheritance in the hybrids. The markers are selected based on the task at hand. For example, for determining parental chromosomal contributions, the loci where parental lines are homozygous but different are appropriate. If the task is to determine recombination rates and frequencies, the appropriate markers are the loci where one or both the parental lines are heterozygous.

In either case, the starting point is a Single Nucleotide Variant (SNV) caller that is suitable for aneuploid genomes. Traditional SNP callers are mainly designed for diploid genomes and work poorly on non-disomic chromosomes. To address this issue, PAINT hosts a custom SNV caller that extracts heterozygous SNVs based on the somy of the chromosome because somy dictates the frequency of heterozygous alleles. For example, a disomic chromosome contains heterozygous alleles each with 50% frequency, a trisomic chromosome contains one allele at 33% frequency and the other allele at 66%, and a tetrasomic chromosome contains one allele at 25% and the other allele at 75% or both alleles at 50% frequency. Therefore, variable thresholds must be employed for individual chromosomes based on their somy. Existing SNV callers mostly employ global thresholds determined using the overall distribution of the data. The cases where heterozygous SNVs are scarce, creating a representative data model is not possible, and when genomes are aneuploid, due to an averaging affect, the data model does not adequately accommodate nondisomic chromosomes.

The SNV caller in PAINT addresses these issues by employing thresholds individually and chromosome-wise based on their somies. The appropriate markers are selected from the SNV files and nucleotide compositions and their frequencies are determined from all the samples. These summary files called “Merged Allele File” and “Merged Frequency File” serve as inputs to the utilities in PAINT that determine parental chromosomal inheritance and calculate recombination rates and frequencies. PAINT is compatible with vcf format outputs from other SNP callers offering additional flexibility to the users in case they wish to employ their favorite tool.

### 2.2. Determining Ploidies

**Figure 3:**
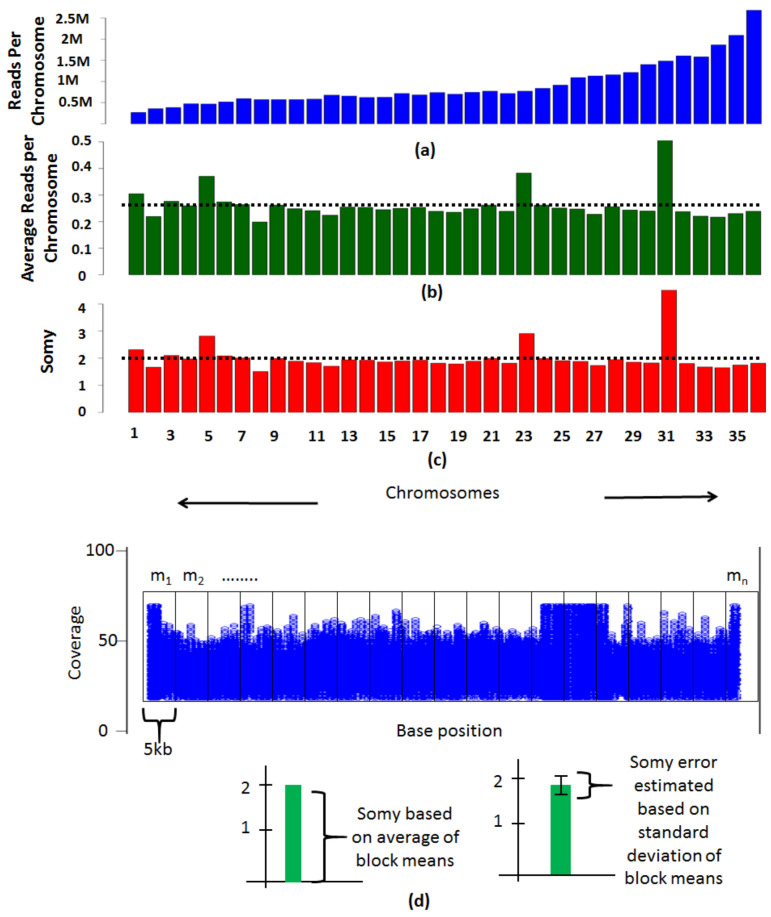
Methods to determine somies. (**a**) The reads per chromosome are determined by counting the number of reads that align to each chromosome. (**b**) Average reads per chromosome is calculated by normalizing the coverage values by the size of the chromosome. The mean of average reads as indicated by the dotted line is suggestive of the overall ploidy of the organism. (**c**) The average read alignment values are scaled to the ploidy of the organism to determine the somies. (**d**) Each chromosome is divided into blocks of 5 kb and average coverage within each block is determined. The average of block means is scaled to the overall ploidy of the organism determined using total DNA content to infer the somy of the chromosome. The standard deviation of the block means is scaled to the ploidy of the organism to determine the method error in estimation of the somy. A second stage scaling is performed by selecting chromosomes with somy close to the ploidy of the organism and finding their average. This average is reflective of the overall ploidy of the organism. The chromosomes are rescaled accordingly. The variation of means from each block across the chromosome is reflective of possible somy values a chromosome can take.

Determining the ploidies involves quantifying the reads that align to each chromosome. The number of reads that align to each chromosome are dependent on two factors, the length of the chromosome and the number of copies of the chromosome. By eliminating the effect of the length of the chromosome, the number of copies (somy) of each chromosome can be determined. The noisy genomic regions such as nucleotide repeats, or gene families often have elevated coverages and must be removed prior to ploidy estimations. PAINT follows a genome-feature agnostic procedure to eliminate them by filtering regions that are greater than twice the average coverage or less than half of the average coverage of the chromosome. Alternate thresholds that are appropriate for the genome under consideration can be chosen by the user or no thresholds may be applied based on individual preference. A simpler method for determining somies is based on read counts to each chromosome, which is more accurate for genomes not sequenced at a higher coverage. Bigger chromosomes have more reads aligning to them in comparison to the smaller chromosomes (Figure 3a). The read counts per chromosome are normalized to the length of the chromosome to nullify the effect of chromosome size. The average of values that are close to the dotted line (Figure 3b) must be scaled to the ploidy of the organism to reveal the somies (Figure 3c). Since everything is relative, the chromosomes with higher or lower values in comparison to the dotted line are scaled accordingly. A more thorough method follows a block-based strategy where each chromosome is divided into blocks of a user defined size (e.g., 5 kb each) and the variation across the chromosome is determined (Figure 3d).

On an average, dividing a chromosome into blocks of 50 or more yielded comparable results in our datasets. The average coverage values (*m_i_*) for each block is calculated and in order to estimate somy of a chromosome, the average of the block coverages is found for each chromosome (*A_c_*) using the following formulae.

$$A_{c}=\frac{m_{1}+m_{2}+\ldots +m_{n}}{n},\;\;A_{s}=\frac{A_{c1}+A_{c2}+\cdots +A_{ck}}{k},\;\;S_{ci}=\frac{\sum {\left(A_{ci}-A_{s}\right)}^{2}}{n-1}$$

Here, *A_c_* is the average coverage of means of ‘*n*’ blocks within a chromosome, *A_s_* is the average of *A_c_* of all the *k* chromosomes (average genome coverage can also be used here) and *S_c_* is the standard deviation of coverages across all the blocks for each chromosome, where subscript “*i*” refers to each individual block. The value *A_s_* is employed to normalize the *A_c_* values and scaled to the organism ploidy to determine chromosomal somies. Selective scaling may be performed to fine tune the somy values by ignoring the aneuploid chromosomes that are not true representatives of average ploidy of the organism. The variation across the chromosome allows assessing error in calculation of somies. This is estimated by leveraging the standard deviation of block means (*S_C_*) which when scaled to the ploidy of the organism provides a measure of error in estimation of somies.

### 2.3. Finding Single Nucleotide Variations (SNVs) and Determination of Parental Contributions

PAINT takes read alignments to the genome as input and generates an allele file that contains nucleotide composition (A, T, C, G) and their frequencies at each genomic position. This is a tab-delimited file with columns indicating genomic position, nucleotide composition, read coverage, frequencies of nucleotides (A, T, C, G), number of forward reads, number of reverse reads, and average mapping quality. The user chooses minimum coverage and minimum allele frequency determined using somies to call nucleotide compositions. We define the term major allele as the allele that is present on the haplotype with highest copy number and minor allele as the allele on the other haplotype. For diploid organisms, heterozygous allele frequencies are close to 50%, for triploid organisms, the minor allele occurs with ~33% and the major allele with ~66% frequency, and so on. Sequencing, genome composition, misalignment, population-based artifacts, and other experimental artifacts can be filtered out to some extent by this initial screening. If at any given position there are no alleles that meet the chosen criteria, it was reported as missing data (−). For example, if the chromosome with maximum somy in the dataset happens to be 4, one can expect a minor allele to occur at 25% frequency and therefore a frequency threshold of 0.15 or more for the minor allele and 0.85 or less for the major allele are appropriate to call heterozygous alleles. By ignoring some loci which do not meet the inclusion criteria, parental inheritance in hybrids can be determined by using only those loci with high confidence.

PAINT implements a custom-built algorithm to identify SNVs using the alleles and their frequencies that are compared against a reference file in FASTA format. The parameters of this SNV caller can be adjusted for haploid, diploid, or aneuploid genomes. The homozygous nucleotides that are different from the reference allele are tagged as homozygous SNVS. For calling the heterozygous SNVs, PAINT first quantifies the somies of each individual chromosome and determines the thresholds based on the somy.

PAINT allows the user to adjust the parameters by which this is done. In our studies we have employed the following: For chromosomes with somy 2, each allele ideally occurs 50% of the time (1/2 = 0.5) and therefore the heterozygous alleles must each have an experimental frequency close to 0.5. Similarly, a trisomic chromosome will have minor allele with 33% frequency (1/3 = 0.33) and major allele with 66% frequency (1 − 1/3 = 0.66). The thresholds for chromosomes with other somies can be determined in a similar fashion. As desired, users may employ parameters from other conceptualizations.

The user can choose the minimum coverage acceptable for calling an allele, and for paired-end data, PAINT ensures considering genomic segments where reads align to the genome in forward and reverse orientations. The program outputs standard information such as chromosome, position, reference allele and alternate allele in tab-delimited or standard variant call format (vcf). The markers that are employed for finding the parental contributions in the hybrids are the homozygous SNV differences between the parental lines. Homozygous SNVs common to both the parental lines are filtered out as these usually represent loci where both the parental lines are the same but are different from the reference genome. The remaining loci represent SNV differences between the parental lines which are used as markers to calculate the parental contributions. PAINT tabulates the nucleotide composition of the hybrids at these markers and generates a “Merged allele file”. This file can be further formatted using PAINT into other formats suitable as inputs for tools, such as for generating linkage maps and for GWAS-based analyses.

## 3. Results

### 3.1. Demonstration of PAINT Pipeline Using Simulated Datasets

Three different simulated datasets highlight different aspects of the PAINT pipeline. Dataset1 highlights the challenges in somy determination at low read coverages. Dataset2 is designed to highlight the preprocessing by PAINT pipeline to facilitate recombination detection by softwares that generate genetic maps. Dataset3 highlights the challenges in dealing with hybrids between two different species. Dataset1 is constructed by sampling a high coverage *L. major Friedlin FV1* data at 27× coverage [31] and down sampling it at various low coverages. This is not a true simulated dataset but rather real data that has been down sampled to mimic datasets that are routinely sequenced at lower coverages. Dataset2 is a simulated dataset built by generating a reference genome containing 3 random sequences of 150 k, 300 k, and 450 kbps respectively. Parent 1 is generated by introducing a total of 140 SNVs (46 homozygous and 96 hetreozygous at ~1 SNV/6.5 kb) randomly into the reference genome. Similarly, parent 2 is generated by introducing 900 SNVs (450 homozygous and 450 heterozygous ~1 SNV/kb) into the reference genome. Crossovers are randomly introduced between the homologous chromosomes from the parental lines at the loci as shown in Table 1 with each chromosome having a cumulative of 4 recombinations in 6 hybrids. The parental chromosomes are randomly assorted and selected to generate diploid hybrids containing disomic chromosomes. A partial loss of heterozygosity (pLOH) is introduced into chromosome 1 of hybrid 2 to mimic partial contribution by parent 2. Total loss of heterozygosity (tLOH) is introduced into chromosome 2 of hybrid 1. The NGS reads for parents and hybrids in dataset 2 are simulated using wgsim package at 30× coverage [33]. Wgsim is a software package that generates short reads that are like those generated by short read sequencers such as Illumina Hi-seq machine. The simulated dataset 2 represents data where the hybrids have undergone a traditional meiosis. This represents a simple genome which does not contain mosaic aneuploidy or artifacts such as repeated elements and gene families which pose serious challenges in alignment and downstream analyses. Dataset 3 is not a truly simulated data and is generated by employing the first five chromosomes from *L. major* v.6.0 and *L. infantum* v.4.2 reference genomes [30,31]. The five chromosomes from each of the parental lines are modified by introducing SNVs randomly into the chromosomes. Parent 1 is generated by introducing 1 SNP/5 kb and parent 2 is generated by introducing 1 SNP/0.6 kb randomly into their respective homologous chromosomes. Both the parental lines are simulated to be diploids with all the chromosomes having disomic profile. The hybrid chromosomes are generated by random segregation and selection of parental chromosomes in different ratios as shown in Table 2. The NGS reads are simulated for each of the parents and hybrids using wgsim package at 15× coverage [33].

### 3.2. Determination of Somies Using NGS Alignments

**Figure 4:**
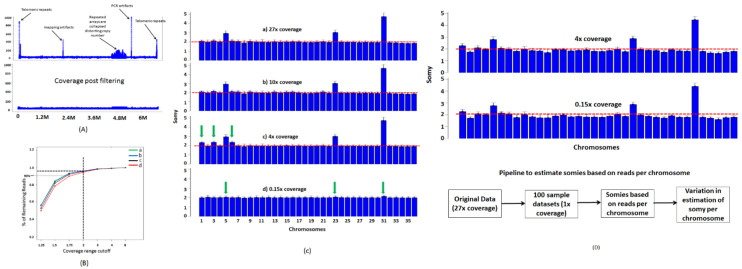
Somies determined using a block-based strategy. (**A**) Coverage plot of a representative chromosome LmjF.12 from dataset 1 shows some artifacts that are commonly seen during read alignments. All regions with coverages twice or half of the average coverage of the genome are removed. (**B**) Rationale for choosing 2 as a range cutoff is based on a threshold selection that retains more than 90% of the reads. Four *L. major Friedlin* datasets from short read archive SRR1028167 (**a**), ERR013300 (**b**), ERR876025 (**c**), and ERR143186 (**d**) were aligned to *L. major Friedlin FV1* genome V. 6. We applied various thresholds between 8 and 1.25 to determine % of reads that are filtered out at each threshold. A threshold of 2 retained more than 90% of the reads and was chosen as the cutoff to eliminate regions with artifacts from the genome. (**C**) The somy determination using block average method using dataset 1 fails at extremely low coverages. At high coverages (>10×), samples indicate that most chromosomes in the *L. major Friedlin* genome are disomic but deviation from disomy is clearly seen in chromosomes 5, 23, and 31. At low 4× coverage, error in estimation of somy increases resulting in some chromosomes showing artificial elevation in somy, indicated by green arrows. At very low coverage (<1×), neither the variations across the chromosome nor the average coverage can be accurately assessed. (**D**) Somies can be estimated at extremely low coverages using reads aligned per chromosome. To estimate the error in estimation of somy for each chromosome, we sampled the original 27× coverage dataset at 1× coverage and generated 100 random datasets. We calculated the error in estimation of somy per chromosome by calculating variation of each chromosome across the 100 datasets. On average, the estimated somies were within an error of 5.5% of initially estimated somies using high coverage data.

The read coverages across the genome are not uniform but vary depending on the genome composition. While most of those variations depend on nucleotide compositions, there are certain elements such as repeats and gene families (Figure 4A) which over represent and bias the coverage statistics. The effects of these noisy elements must therefore be removed for reasonable estimation of somies. We sought to find a reasonable coverage threshold to eliminate these regions using 4 random samples and using various threshold ranges. We randomly selected 4 *L. major* datasets from short read archive and aligned them to *L. major Friedlin FV1* 6.0 genome. We sought to find the maximum threshold at which we could eliminate the noisy regions while retaining more than 90% of the aligned reads. We applied various threshold ranges between 1.25 and 8 and eliminated regions that did not qualify these threshold ranges and counted the remaining reads. At a liberal threshold of 8, not all the intended noisy regions were filtered out but retained more than 99% of the reads (Figure 4B). At threshold <2, all noisy regions were eliminated but less than 90% of the aligned reads were retained. We found that a threshold of 2 provides us with a good balance between removing noisy regions and retaining more than 95% of the reads. After removing the noisy segments, we divided each chromosome into blocks of 5 kb and found average coverage within each block. Based on these criteria, we found that all chromosomes except for chromosomes 5, 23, and 31 are disomic, as indicated by the red horizontal line (Figure 4C(a)).

The method based on block averages performs well for datasets with high coverage (Figure 4C(a,b)). However, at low coverages, the sparseness results in inadequate representation of certain regions in the genome, causing inaccurate estimation of somies. To illustrate this concept, we randomly sampled the original dataset (27× coverage) at various sampling rates to generate bootstrapped datasets at various coverages. At 10× coverage, the estimated somies were similar to the somies estimated at high coverage. (Figure 4C(b)). At 4× coverage, although the somies can be accurately estimated for majority of the chromosomes, noise starts to creep in as indicated by the arrows (Figure 4C(c)). We also found this phenomenon at other sampling rates between 1× and 5×. We finally estimated somies using the blocks method at less than 1× coverage. At these extremely low coverage values, most of the genome is not covered and the regions that are covered have average coverage of 1 (Figure 4C(d)). Therefore, the blocks method cannot effectively capture the aneuploidy.

To address the issue of low coverage, which can occur in situations where host DNA, e.g., spleen, is much higher in comparison to parasite DNA, we followed the method based on read counts. The estimated somies at 4× coverage reveals somies similar to those found using high coverage datasets (Figure 4D). At extremely low coverages (<1× coverage), the results are still consistent with high coverage data. The variation across the chromosome is not an accurate measure of error in estimation of somies at low coverages. Therefore, we estimated the errors by measuring variations in somies of 100 bootstrapped datasets at 1× coverage and calculating intrachromosomal somy variation across all the bootstrapped datasets. The estimated somies were on an average within 5.5% error of somies calculated using the original dataset (27× coverage). We repeated the experiment using much lower coverages such as 0.5× and 0.15× and obtained similar results. We repeated the entire experiment using *L. major LV39* and *L. infantum* NGS datasets and obtained similar results.

### 3.3. Determination of Parental Inheritance

**Figure 5:**
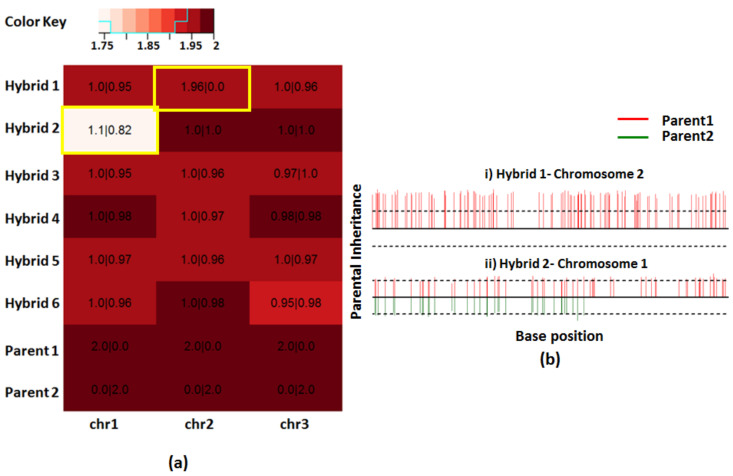
Determination of parental inheritance using simulated dataset 2. (**a**) Heatmap of the ploidies for the simulated hybrids indicates that most chromosomes are near disomic. Overlaid on the top is the number of chromosomes inherited from each parental line in the M|N format where “M” and “N” are the number of chromosomes inherited from parents 1 and 2, respectively. Most hybrid chromosomes show balanced contribution from each parent. Highlighted are the two chromosomes which had partial or no contribution from one of the parents. (**b**) Bottle brush plots showing the two chromosomes with unbalanced parental contributions. Only parent 1 contributed to chromosome 2 in hybrid 1, resulting in total LOH (loss of heterozygosities). Parent 2 has partially contributed towards chromosome 1 in hybrid 2, resulting in partial LOH.

Simulated dataset 2 allowed us to incorporate features that can be verified against the results obtained using the computational pipelines. The homozygous SNVs are used as markers to study the inheritance of parental chromosomes in the hybrids. The heatmap of the somies indicated that the estimated somies for most chromosomes were between 1.95 and 2 indicating an error of a maximum 0.05 somy units. We simulated chr. 1 of hybrid 2 and chr. 2 of hybrid 1 to have loss of heterozygosities (LOH). The heatmap clearly shows chr. 1 of hybrid 2 to contain less somy indicating a possible partial LOH (Figure 5a). Overlaid on the heatmap is the parental chromosomal inheritance values determined using the average of these values calculated at markers across the chromosome. Markers where parental lines are homozygous different allow one to calculate allele frequencies of parental alleles in the hybrids. These allele frequencies when multiplied by the chromosomal somies reveal parental chromosomal inheritance. The parental inheritance at each marker can also be drawn allowing one to study the extent of LOH (Figure 5b). These “bottle brush” plots indicate that chr. 2 of hybrid 1 had a total LOH of parent 2 and chr. 1 of hybrid 2 had a partial LOH. Heatmaps provide a global picture of aneuploidy of all the samples under study. The parental inheritance overlaid on the top allows one to accurately assess the contributions made by each parent. The “bottle brush” plots allow studying interesting chromosomes at high resolution and for pinpointing the start of a LOH on individual chromosomes.

### 3.4. Recombination Detection Using Genetic Maps

**Figure 6:**
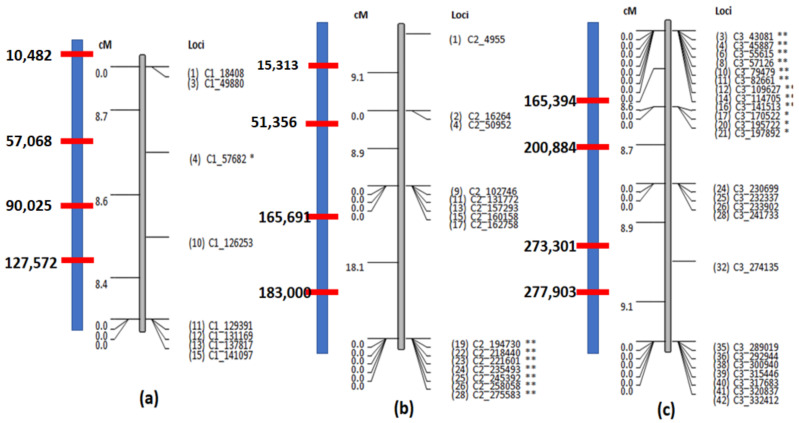
The linkage maps of the three chromosomes using simulated dataset 2. (**a**) Chromosome 1 has only a few markers which could capture only 3 out of the 4 recombinations introduced. The recombination on hybrid 4 at 10,482 (Table 1) could not be captured as there are no markers before 18,408. (**b**) All the 4 recombinations introduced into chromosome 2 are captured. However, since the markers are unevenly distributed, two recombinations between 162,758 and 194,730 cannot be accurately pinpointed. (**c**) All the 4 recombinations introduced into chromosome 3 can be more accurately pinpointed because of the presence many markers.

Genetic maps provide a visual representation of recombination hotspots captured using heterozygous markers that are a consequence of differences among homologous chromosomes. In *Leishmania*, it is difficult using WGS data to deconvolute the markers that are a consequence of homologous differences from those that occur due to mosaic aneuploidy. Moreover, the isolates we studied have low heterozygosity, compounding the problems associated with mosaic aneuploidy. But for organisms that do not exhibit mosaic aneuploidy, this module in PAINT can be leveraged as demonstrated using simulated dataset 2. The loci where the parental lines are heterozygous, the hybrids can be either heterozygous or homozygous depending on random segregation, selection and recombination. The markers before and after the recombination breakpoints introduce minute changes in the genetic profile that can be translated into centimorgan distances. PAINT takes the markers where one or both of the parents is heterozygous and captures the genetic profile of the hybrids at those loci. The “merged allele” file that is generated can be transformed into various formats including the mapmaker format [34] that is widely accepted by genetic map drawing softwares such as mapdisto [35]. The centimorgan distances from the linkage map for the simulated hybrids from dataset 2 obtained using mapdisto are compared against the true recombinations introduced into the hybrids (Table 1). The results indicate that the recombinations can be accurately pinpointed at various resolutions depending on the number of markers and their distribution across the chromosome (Figure 6). Chromosome 1 has 4 recombinations introduced into different hybrids, 3 of which were captured using the genetic maps. The recombination at 10,482 on hybrid 4 could not be captured as there are no markers before 10,482 on chromosome 1 (Figure 6a). The 4 recombinations introduced into chromosome 2 were detected but the recombinations at 165,691 and 183,000 could not be accurately pinpointed because of the resolution of the markers and their placement (Figure 6b). All of the 4 recombinations in chromosome 3 could be accurately pinpointed because of the presence of high-resolution markers (Figure 6c).

### 3.5. Analysis of Interspecies Hybrids

Crosses between different species in higher order organisms is not very common but in *Leishmania*, this is routinely seen. PAINT pipeline for the interspecies hybrids focuses only on the conserved regions across both the species. We devised a strategy to extract conserved regions based on the alignments of the reads to genomes of both the species. The reads from the first parental line are aligned to the reference genomes of both the species. The conserved reads that align to both the genomes are retained. Independently, the reads from the other parental line are aligned to genomes of both the species, and again the conserved reads are retained. The conserved regions from both alignments are compared, the regions common to both are retained and the analysis is restricted to these regions. Since the idea is to find regions of the genome that are more or less conserved across both the genomes, and since the homozygous SNPs are employed for downstream analyses, the choice of alignment algorithm or the parameters employed for alignment have minimal effect.

**Figure 7:**
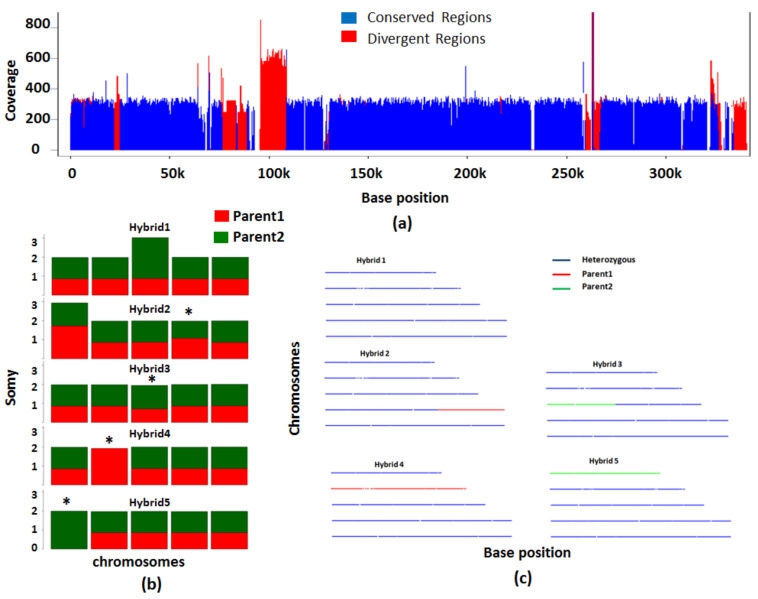
Analysis of interspecies hybrids using simulated dataset 3. The analysis of hybrids from parental lines belonging to two different species focuses on conserved regions between the two species. (**a**) The conserved regions between the two species on Chromosome 2 are shown in blue. The regions that are not conserved are shown in red. (**b**) The bar plot shows chromosomes inherited from each of the parental lines. The chromosomes with loss of heterozygosity (LOH) are indicated by asterisk. (**c**) Line plots show the heterozygous nature of each chromosome in the 5 hybrids. The hybrid chromosomes are indicated in blue. In red or green are the chromosomes which have contribution only from only one parent.

Simulated dataset 3 is used to illustrate the efficacy of PAINT in analyzing hybrids corresponding to two *Leishmania* species, *L. major* and *L. infantum*. The composition of different hybrid chromosomes in the 5 simulated hybrids is shown in Table 2. The reads that are aligned to both the genomes are considered conserved (Figure 7a, shown in blue) and the divergent regions shown in red are not considered for further analysis. Using the markers that are homozygous and different, the inheritance of the parental chromosomes in the hybrids is found. Barplots indicate the contributions of parental lines in the hybrids depicting aneuploid chromosomes as well those with LOH (marked by * Figure 7b). The disomic chromosomes with a total LOH contain only one color corresponding to the parental line that has contributed to the hybrid. The disomic chromosomes with partial LOH show colors corresponding to both the parental lines but a difference in the height of the bars is seen. The aneuploid chromosomes such as chr. 3 of hybrid 1 show extra chromosomal contribution made by parent 2. The line plots show a snapshot of the heterozygosity and the LOH of the hybrid chromosomes (Figure 7c). Hybrid 1, for example, is a complete hybrid (shown in blue) with contributions from both the parental lines across all 5 chromosomes, while hybrids 2 and 3 are complete hybrids except for a partial LOH confined to one chromosome, and hybrids 4 and 5 are also complete hybrids except for a total LOH in one chromosome. Barplots are good at assessing aneuploidy and relative parental contributions, and line plots help in painting global zygosity profiles.

### 3.6. PAINT Supports Meiosis-Like Sexual Recombination in Experimental Hybrids

We previously employed PAINT to study intra and interspecies hybrids in *Leishmania* by analyzing high-resolution WGS data [31]. We determined the somies and chromosome inheritance patterns of 44 hybrids generated within and between different old-world species of *Leishmania*, including *L. major*, *L. infantum*, and *L. tropica*, and compared them against the inheritance patterns expected under meiosis. In each case, the hybrids appeared equivalent to F1 progeny, heterozygous throughout most of the genome for the markers that were homozygous and different between the parents. The parental contributions in the hybrid chromosomes matched the ratios expected under meiosis 97–99% of the time. Most of the progeny were near diploid and showed balanced segregation of the chromosomes where both parents were disomic. Trisomic chromosomes in the parents were passed on to the progeny as single or double copy in frequencies expected by Mendelian segregation. Tetrasomic chromosomes were passed on in double copy and not in quadruple copy. The instances of non-Mendelian inheritance patterns that might support a parasexual process were rare (<3% of chromosomes) and were presented as a gain of somy in comparison to either parent, and uniparental inheritance that we interpret as a loss of heterozygosity that occurred during meiosis or subsequent mitotic generations.

The most compelling argument in favor of a meiotic process, revealed using PAINT, is the pattern of recombinations observed in the backcrosses generated from experimental and natural hybrids. The analysis of the backcross progeny revealed discrete blocks of homozygous and heterozygous SNPs throughout the genome, consistent with meiotic recombination of the homologous chromosomes in the hybrid parent. The recombinations obtained using the backcross progeny allowed us to construct the first physical map of recombinations in *Leishmania*. For our experimental crosses, the average recombinations for *L. major* and *L. tropica* was 1 cM per 17,391 bp and 15,300 bp, respectively, which compare well with the 1 cM per 24,406 bp recombination frequency reported for *Trypanosoma brucei* [36]. These recombination frequencies are more than adequate for linkage analyses. Altogether, the WGS analysis of experimental hybrids has provided the clearest evidence to date of meiosis-like sexual recombination in *Leishmania*.

Several variations of meiosis have already been described in other organisms that can accommodate aneuploid genomes. Meiotic chromosome segregation is well described in triploid strains of *Saccharomyces cerevisiae* which accurately produce viable tetrads containing 2 spores with 2 copies and 2 spores with 1 copy of each homolog [37]. Furthermore, all 3 copies of a trisomic chromosome in a close to diploid strain of *S. cerevisiae* were shown to undergo recombination in a single meiosis [38]. In Drosophila, although the fertility of triploid females is reduced, viable offspring between diploids and triploids can be obtained [39]. Finally, we refer to the sexual strategy as meiosis-like because so far we have no direct evidence for haploid (or close to haploid) intermediate stages in *Leishmania*, and other sexual processes are possible. In *Saccharomyces*, fusion of parental diploid cells followed by meiosis, termed tetraploid meiosis, results in fusion of haploid nuclei to form diploid progeny [40]. The fact that a few experimental tetraploid hybrids have been recovered from sand flies supports that the fusion of diploid cells can occur and that a tetraploid immediate stage may exist.

## 4. Conclusions

Three different simulated datasets were presented that focus on different aspects of the PAINT framework that can be employed to analyze, interpret, summarize and paint experimental hybrids profiled using WGS platforms. Dataset 1 was used to illustrate challenges associated with somy determination at various read coverages. The strategy based on average block coverages is adequate and preferred to assess the somies at high coverages. However, at low coverages, this method is insufficient and alternate strategies based on reads per chromosome are needed. The homozygous SNV differences between the parental lines were employed to reveal parental contributions in the hybrids and in unwrapping events such as LOH. The features introduced into the simulated dataset such as aneuploidy, LOH and recombinations compared favorably against the results obtained using PAINT. Dataset 2 was used to highlight how the PAINT pipeline can facilitate recombination detection by genetic map generating software. The genetic maps based on the resolution of the parental heterozygous SNVs indicate that regions with high centimorgan distances coincide correctly with the known recombinations introduced into the hybrids. Dataset 3 demonstrated the analysis of interspecies hybrids by focusing on the regions that are conserved between the two species.

We applied PAINT to the analysis of high-resolution WGS data to understand the mode of sexual reproduction employed by experimental *Leishmania* hybrids. We designed PAINT to reveal somies with accuracy using multiple strategies and by filtering out regions that represent noise. We employed single nucleotide differences between parents to determine their contributions in the hybrids, and to visualize features such as LOH and in pinpointing recombination break points in the backcrosses. All the results indicate that the system of genetic exchange is Mendelian and involves meiosis-like sexual recombination. The recombinations obtained using backcrosses and revealed using PAINT show that the tools are available for linkage studies and positional cloning of important genes. PAINT is a java-based utility that requires no installation and can be obtained freely from http://ageless.sourceforge.net/. Since high demanding computations are performed outside the PAINT pipeline, the pipeline can be executed even on computers with basic configuration such as 512 Mb RAM and 2.5 Ghz processor.

## Figures and Tables

**Table 1:** Recombination loci in the hybrid chromosomes introduced into Simulated data 2.

	Chr. 1	Chr. 2	Chr. 3
Hyb. 1	127,572	-	273,301
Hyb. 2	-	165,691	277,903
Hyb. 3	57,068	15,313	-
Hyb. 4	10,482	-	200,884
Hyb. 5	-	183,000	165,394
Hyb. 6	90,025	51,356	-

**Table 2:** Parental inheritance in the hybrid chromosomes in simulated data 3.

	Hybrid 1	Hybrid 2	Hybrid 3	Hybrid 4	Hybrid 5
Chromosome 1	1:1	2:1	1:1	1:1	0:2
Chromosome 2	1:1	1:1	1:1	2:0	1:1
Chromosome 3	1:2	1:1	0.5:1	1:1	1:1
Chromosome 4	1:1	1:0.5	1:1	1:1	1:1
Chromosome 5	1:1	1:1	1:1	1:1	1:1

## Data Availability

PAINT is available for download from http://ageless.sourceforge.net/.
